# A Novel Host-Based Immunotherapy for the Suppression of HBV and HCV Replication: Heat-Killed *Caulobacter crescentus* (HKCC)

**DOI:** 10.3390/cells15131172

**Published:** 2026-06-27

**Authors:** Raj S. Patel, Nancy Gupta, Satish Vedi, Rakesh Kumar, Babita Agrawal

**Affiliations:** 1 Department of Surgery, Faculty of Medicine and Dentistry, College of Health Sciences, University of Alberta, Edmonton, AB T6G 2S3, Canada; rsp@ualberta.ca; 2 Department of Laboratory Medicine and Pathology, Faculty of Medicine and Dentistry, College of Health Sciences, University of Alberta, Edmonton, AB T6G 2S3, Canada; gupta1@ualberta.ca (N.G.); satish.vedi@gmail.com (S.V.); 3 ImMed Biotechnologies, Edmonton, AB T6R 2E8, Canada; rakesh.rkumar1988@gmail.com

**Keywords:** hepatitis viruses, immunotherapy, postbiotic, antiviral immunity, hepatitis B virus, hepatitis C virus

## Abstract

**Highlights:**

**What are the main findings?**

- Heat-killed *Caulobacter crescentus* (HKCC), a non-infectious bacterium, triggers a broad, multi-functional cytokine response in human peripheral blood mononuclear cells (PBMCs).
- Stimulated immune cell supernatants—alone or with antivirals—strongly suppress both HCV and HBV replication without cytotoxicity.

**What are the implications of the main findings?**

- HKCC targets the host immune system, offering a way to circumvent the development of drug-resistant virus strains.
- Combining this immunotherapeutic with antivirals could shorten treatment times and stop viruses from rebounding or reactivating.

**Abstract:**

**Background:** Hepatitis B and C viral infections remain a significant global health challenge, despite the implementation of an effective direct-acting antiviral (DAAs) and nucleos(t)ide analogues (NAs). Current HBV therapy is not curative as stopping therapy usually leads to active disease in most patients requiring long-term treatment. Although current HCV-DAAs are highly effective they fall short due to arising drug-resistance and have limited ability to avert re-infections. Furthermore, current HCV DAA treatments lead to the reactivation of occult HBV infection, compromising the effectiveness of current antiviral therapies, and increasing the risk of severe liver complications like cirrhosis and hepatocellular carcinoma. In addition, current treatments do not restore the immune dysfunction, a characteristic of chronic HBV infection. Given the global burden of disease, there is an urgent need for more effective therapy that can shorten the duration of treatment and achieve high rates of HBsAg reduction. Combining an antiviral to reduce viral antigen burden and an immunomodulator to boost the immune response could provide an effective treatment for HBV/HCV infections. **Methods:** In this study, we explored the potential of a novel bacterial therapeutic agent, heat-killed *Caulobacter crescentus* (HKCC), as an alternative and/or adjunct host-based therapy for HCV and HBV infections. Here, we have investigated the antiviral effects of the HKCC-stimulated human PBMCs using in vitro HCV and HBV infection models to assess viral replication, viral relapse responses, protein expression, and cytotoxicity. **Results:** Our findings reveal that HKCC induced a multi-functional cytokine response (IFN, TNF, IL-2, IL-10, IL-6, IL-17A, and IL-22) in PBMCs obtained from multiple healthy donors. Supernatants collected from these HKCC-stimulated human PBMCs, alone and in combination with antivirals, strikingly inhibited HCV replication and viral relapse responses without inducing any cytotoxic effects on HCV-1a replicon cells. In addition, these PBMC supernatants, with or without antivirals, led to the suppression of HBV DNA replication and inhibited HBsAg and HBeAg production in HepG 2.2.15 cells. **Conclusions:** In conclusion, HKCC is a promising candidate for eliminating HBV and HCV infections, and warrants further investigation to potentially contribute to the development of a novel host-based immunotherapy.

## 1. Introduction

Infections with Hepatitis B (HBV) and C (HCV) viruses are a major cause of cirrhosis and hepatocellular carcinoma (HCC). The World Health Organization (WHO) estimates that more than 250 million people were living with chronic HBV in 2022 and more than 1.2 million new infections occur each year, worldwide resulting in an estimated 1.1 million deaths [1]. Globally, around 50 million people have chronic HCV infection with 1.0 million new infections occurring per year [1]. HBV and HCV commonly spread by sexual transmission, perinatal transmission, infected blood, tattooing, needle stick injury, contaminated needles and syringes and exposure to infected body fluids such as saliva, vaginal and seminal fluids [2]. Traditionally, pegylated IFN and ribavirin was the standard treatment for HCV infections, demonstrating a 40–50% cure rate and 49% relapse rate [3]. However, long-term administration of pegylated IFN caused major psychiatric, gastrointestinal, and hematologic side effects. Fortunately, current direct-acting antiviral (DAA) treatments have shown improved efficacy against HCV infections, with a cure rate of 95% and a relapse rate of less than 0.5% with milder side effects [4]. However, treatment outcomes have been negatively affected by (1) the emergence of drug-resistant mutations in HCV genotypes 1 and 3, (2) the adverse effects of drug–drug interactions in combination treatment with various DAAs, and (3) the limited ability to avert reinfections has compromised the effectiveness of HCV DAAs [5,6,7,8,9,10]. Furthermore, HCV-infected patients with occult HBV infections that are treated with current HCV-DAAs have shown to have their HBV infections reactivated [11]. The incidence rate of HCV/HBV coinfection is ~15%; however, 50% of high-risk patients with chronic HCV remain undiagnosed for HBV until after HCV DAA treatment, when HBV infection is reactivated [12]. In contrast to HCV mono-infections, resolving infections in HCV/HBV co-infected patients is more complex and is associated with a greater risk of patients progressing towards cirrhosis and HCC [12]. Therefore, supplementing HCV-DAAs with immunomodulatory therapeutics that can simultaneously control both HBV and HCV replication, their reactivation and selection of drug-resistant mutants, may lead to better treatment outcomes and lower the risk of liver disease in co-infected patients.

Nucleos(t)ide analogues (NAs), including entecavir, tenofovir, and adefovir, with pegylated IFN have been used for treating HBV infection [13]. The current treatments provide long-term control of viral replication, defined by the inhibition of DNA replication and production of Hepatitis B Surface Antigen (HBsAg) and Hepatitis B e-Antigen (HBeAg), but no clearance of covalently closed circular DNA (cccDNA). Therefore, they are not able to eradicate HBV from the liver and have restricted efficacy leading to drug-resistance. Despite life-long NAs administration, HBsAg loss is rare in HBeAg-positive and in HBe-Ag negative patients [14]. Natural resolution of HBV infections has been achieved through the induction of a well-coordinated innate and adaptive immune response, whereas, persistent HBV infections have shown to impair innate triggers, such as the Toll-like receptors (TLRs), retinoic acid-inducible gene I (RIG-I), the apolipoprotein B mRNA editing enzyme catalytic polypeptide-like (APOBEC) proteins, and induced dysfunctional and exhausted CD4^+^/CD8^+^ T cell responses [15,16,17,18]. Recently, studies have shown the earlier induction of IFNγ to degrade HBV cccDNA via APOBEC proteins, highlighting the potential of the innate immune system to resolve HBV infections [19]. HBV infections can be prevented by vaccines, but they are not effective for chronic HBV infections. In contrast, there is no vaccine available for the prevention or treatment of HCV infections. Moreover, vaccine and immunotherapeutic approaches looking to generate antigen-specific immunity against HBV and HCV have been largely unsuccessful due to the virus-induced exhaustion of adaptive immune responses [15]. Thus, immunotherapeutics harnessing the innate immune system may facilitate the revival of exhausted antiviral responses and provide long-term control of HBV and HCV infections.

**Figure 1:**
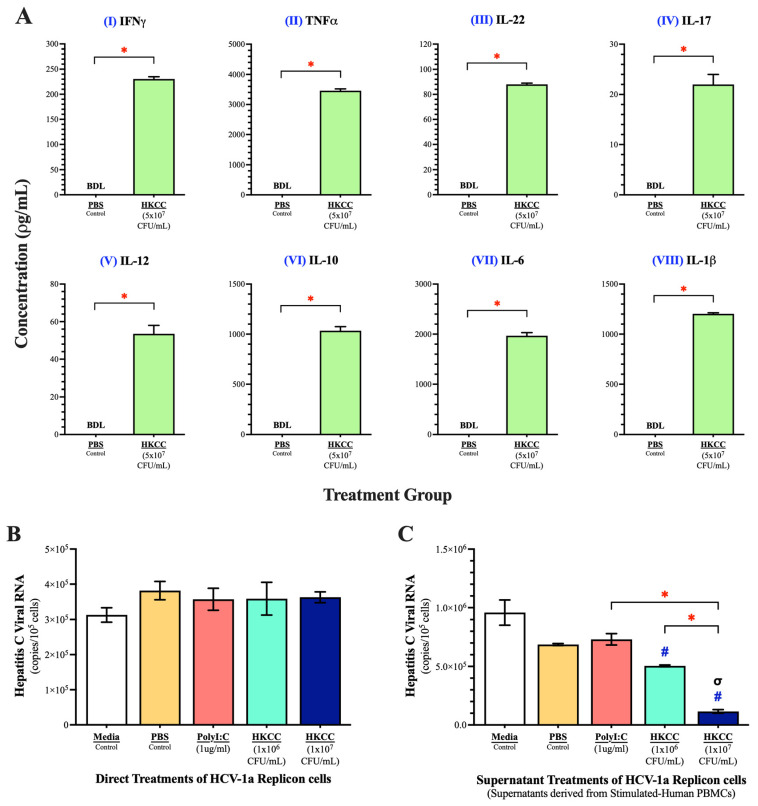
**Heat-killed *Caulobacter crescentus* (HKCC)-stimulated cytokine production in human PBMCs and its supernatant inhibited HCV RNA replication in HCV-1a replicon cells.** (**A**) Human PBMCs (4 × 10^6^ cells/mL; *n* = 12) were stimulated with PBS (white) or HKCC (at 5 × 10^7^ CFU/mL; green) for 24 h. Supernatants were collected and analyzed for various cytokines: (**I**) IFNγ, (**II**) TNFα, (**III**) IL-22, (**IV**) IL-17, (**V**) IL-12, (**VI**) IL-10, (**VII**) IL-6, and (**VIII**) IL-1β, using commercial ELISA kits. (**B**) HCV-1a replicon cells (1 × 10^5^ cells/mL) were treated directly with medium, PBS, polyI:C (1 μg/mL) of HKCC (1 × 10^6^ and 1 × 10^7^ CFU/mL) or (**C**) with supernatants from human PBMCs stimulated with medium (white), PBS (gold), Poly I:C (1 μg/mL; red), or HKCC (at 1 × 10^6^ CFU/mL; aqua), or HKCC (at 1 × 10^7^ CFU/mL; navy blue) for 24 h. HCV RNA levels were quantified by RT-PCR with SYBR. Data were presented as mean ± SEM of four donors from triplicate cultures, from three independent repeat experiments. Statistical differences were determined by one-way, ANOVA followed by Šídák post hoc analysis. Significant differences were indicated between selected treatment groups (*, *p* ≤ 0.05), compared to medium control (**#**, *p* ≤ 0.05), and PBS control (σ, *p* ≤ 0.05).

**Figure 2:**
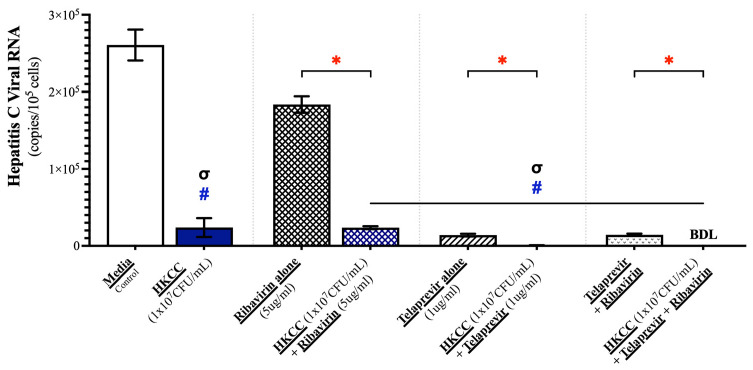
**Combination treatment of HKCC-stimulated human PBMC supernatants with DAAs on HCV RNA replication.** HCV-1a replicon cells (1 × 10^5^ cells/mL) were treated with medium (white), or various DAAs {ribavirin (5 μg/mL; cross-hatched pattern), telaprevir (1 μg/mL; diagonal stripes), telaprevir + ribavirin (3-dot pattern)} alone, and/or in combination with HKCC (1 × 10^7^ CFU/mL)-stimulated PBMC supernatants (navy blue). After one treatment cycle (each treatment cycle is 4–5 days), HCV RNA levels were quantified by RT-PCR with SYBR. Data were presented as mean ± SEM of four donors from triplicate cultures, from three independent repeat experiments. Statistical differences were determined by one-way ANOVA, followed by Šídák post hoc analysis. Significant differences were indicated between selected treatment groups (*, *p* ≤ 0.05), compared to medium control (**#**, *p* ≤ 0.05), and PBS control (σ, *p* ≤ 0.05). BDL = Below detection limit.

**Figure 3:**
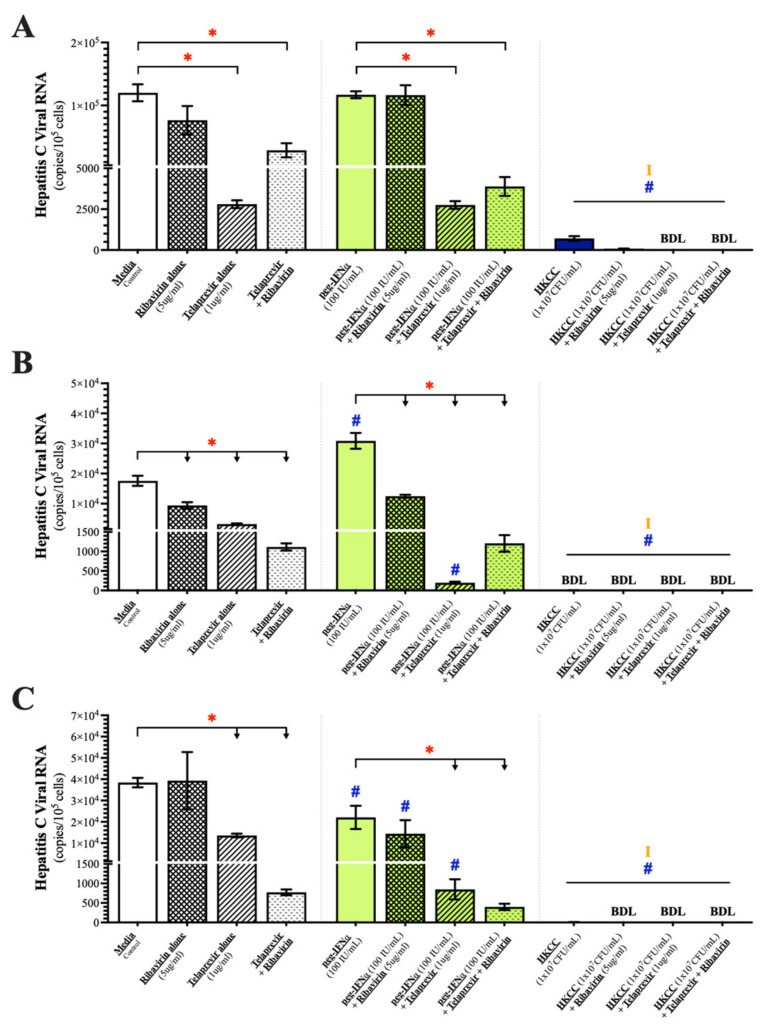
**Combination treatment of HKCC-stimulated PBMC supernatants with DAAs and comparison with peg-IFNα treatments alone, and/or in combination with DAAs on HCV RNA replication.** HCV-1a replicon cells (1 × 10^5^ cells/mL) were treated with medium (white), various DAAs {ribavirin (5 μg/mL; cross-hatched pattern), telaprevir (1 μg/mL; diagonal stripes), telaprevir + ribavirin (3-dot pattern)} alone, and/or in combination with peg-IFNα (100 IU/mL; green) or HKCC (1 × 10^7^ CFU/mL)-stimulated PBMC supernatants (navy blue). After (**A**) two, (**B**) four, and (**C**) five treatment cycles (each treatment cycle is 4–5 days), HCV RNA levels were quantified by RT-PCR with SYBR. Data were presented as mean ± SEM of four donors from triplicate cultures, from three independent repeat experiments. Statistical differences were determined by one-way ANOVA, followed by Šídák post hoc analysis. Significant differences were indicated between selected treatment groups (*, *p* ≤ 0.05), compared to medium control (**#**, *p* ≤ 0.05), PBS control, and peg-IFNα (I, *p* ≤ 0.05). BDL = Below detection limit.

**Figure 4:**
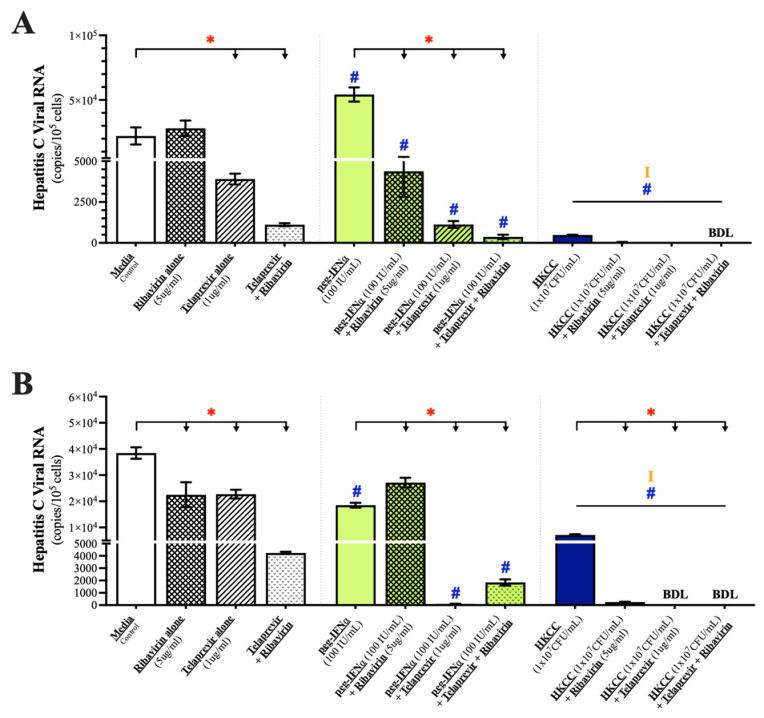
**HCV relapse responses in HCV-1a replicon cells treated with HKCC-stimulated PBMC supernatants or peg-IFNα alone, and/or in combination with DAAs.** HCV-1a replicon cells (1 × 10^5^ cells/mL) were treated with medium (white), various DAAs {ribavirin (5 μg/mL; cross-hatched pattern), telaprevir (1 μg/mL; diagonal stripes), telaprevir + ribavirin (3-dot pattern)} alone, and/or in combination with peg-IFNα (100 IU/mL; green) or HKCC (1 × 10^7^ CFU/mL)-stimulated PBMC supernatants (navy blue). After two treatment cycles (each treatment cycle is 4–5 days), subsequent cultures were left untreated for (**A**) one and (**B**) three rebound periods (each rebound period is 4–5 days), and HCV RNA levels were quantified by RT-PCR with SYBR. Data were presented as mean ± SEM of four donors from triplicate cultures, from three independent repeat experiments. Statistical differences were determined by one-way, ANOVA followed by Šídák post hoc analysis. Significant differences were indicated between selected treatment groups (*, *p* ≤ 0.05), compared to medium control (**#**, *p* ≤ 0.05), PBS control, and peg-IFNα (I, *p* ≤ 0.05). BDL = Below detection limit.

**Figure 5:**
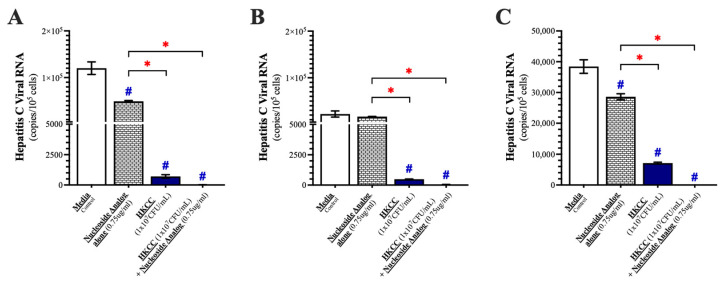
**Combination treatment of HKCC-stimulated PBMC supernatants with nucleoside analog on HCV RNA replication and viral relapse responses.** HCV-1a replicon cells (1 × 10^5^ cells/mL) were treated with medium (white), nucleoside analog (0.75 μg/mL; brick pattern) alone, and/or in combination with HKCC (1 × 10^7^ CFU/mL)-stimulated PBMC supernatants (navy blue) and after (**A**) two treatment cycles (each treatment cycle is 4–5 days). HCV RNA levels were quantified by RT-PCR with SYBR. In a parallel culture, after two treatment cycles, subsequent cultures were left untreated for (**B**) one and (**C**) three rebound periods (each rebound period is 4–5 days), and HCV RNA levels were quantified by RT-PCR with SYBR. Data were presented as mean ± SEM of four donors from triplicate cultures, from three independent repeat experiments. Statistical differences were determined by one-way ANOVA, followed by Šídák post hoc analysis. Significant differences were indicated between selected treatment groups (*, *p* ≤ 0.05), compared to medium control (**#**, *p* ≤ 0.05).

**Figure 6:**
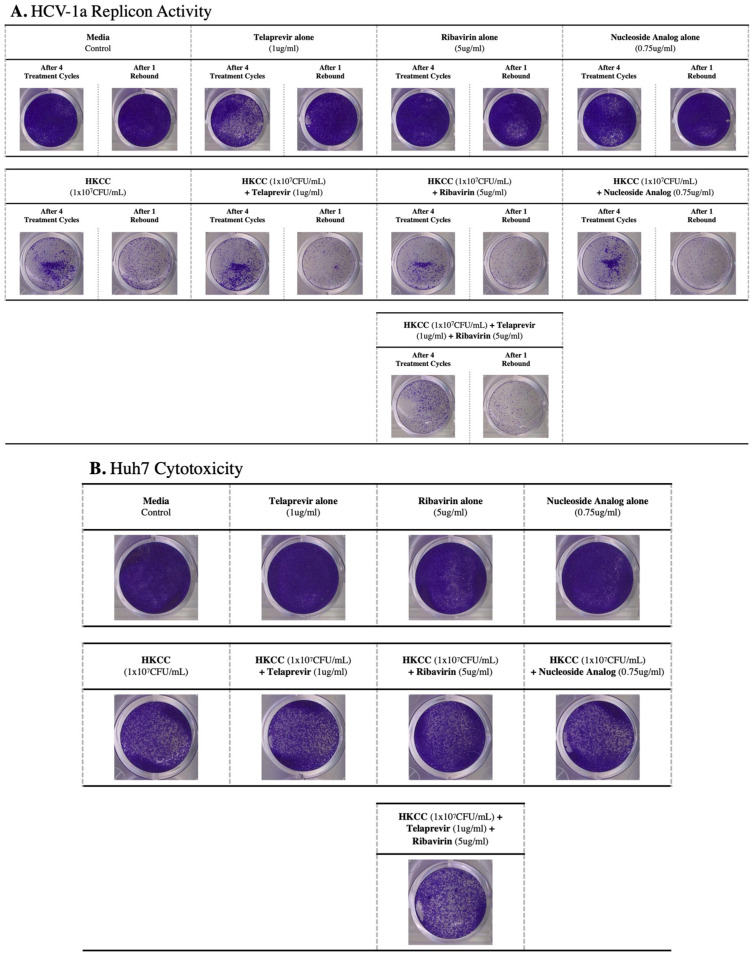
**Crystal violet staining of HCV-1a replicon cells and Huh7 cells after four treatment cycles with various DAAs alone and/or in combination with HKCC-stimulated PBMC supernatants and one rebound period.** (**A**) HCV-1a replicon cells (1 × 10^5^ cells/mL) and (**B**) Huh7 cells (1 × 10^5^ cells/mL) were treated with medium, various DAAs telaprevir (1 μg/mL), {ribavirin (5 μg/mL), nucleoside analog (0.75 μg/mL)} and HKCC (1 × 10^7^ CFU/mL)-stimulated PBMC supernatant alone and/or in a combination of HKCC-stimulated PBMC supernatants with telaprevir, ribavirin, nucleoside analog or telaprevir + ribavirin. Crystal violet staining was performed after the 4th treatment cycle (each treatment cycle is 4–5 days; left). After the parallel culture plates were left untreated for one rebound period (4–5 days; right) and stained with crystal violet. Reduction in crystal violet staining represents inhibition of HCV RNA replication activity, in HCV-1a replicon cells, and increased cytotoxicity, in Huh7 cells.

**Figure 7:**
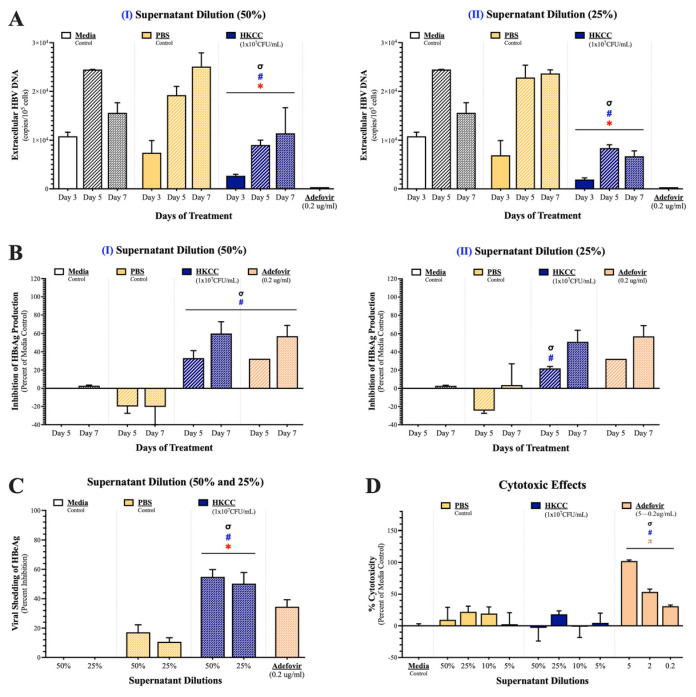
**Inhibition of extracellular HBV DNA, HBsAg, and HBeAg from HepG 2.2.15 cells upon treatment with supernatants from HKCC-stimulated human PBMCs.** HepG 2.2.15 cells (5 × 10^4^ cells/mL) were treated with human PBMC supernatants stimulated with medium (white), PBS (gold), or HKCC (1 × 10^7^ CFU/mL) (navy blue), every 2–3 days, for 7 days (totaling 3 treatment cycles). On the 3rd (solid), 5th (angled stripes), and 7th (angled bricks) day of culture, supernatants were collected to detect the production of (**A**) extracellular HBV DNA, (**B**) HBsAg, and (**C**) HBeAg using RT-PCR and indirect ELISA. Supernatants were tested at two dilutions: (**I**) 50% and (**II**) 25% of the total culture medium. Adefovir (0.2 μg/mL) was used as a DAA positive control. (**D**) Cytotoxic effects of HKCC-stimulated PBMC supernatants (50, 25, 10, and 5%) and adefovir (5, 2, 0.2 μg/mL) were assessed on HepG 2.2.15 cells using XTT assay. Data were presented as mean ± SEM of four donors from triplicate cultures. Statistical differences were determined by one-way ANOVA, followed by Šídák post hoc analysis. Significant differences compared to medium control (**#**, *p* ≤ 0.05), PBS control (σ, *p* ≤ 0.05), Adefovir (*, *p* ≤ 0.05), and HKCC (π, *p* ≤ 0.05) are indicated.

In our earlier studies, we demonstrated that a heat-killed form of a freshwater, non-pathogenic bacterium, *Caulobacter crescentus* (HKCC), acts as a potent innate immune stimulator, inducing antigen-presentation function, and enhancing expression of co-stimulatory molecules and cytokine production from various innate immune cells, including macrophages, dendritic cells, innate lymphoid cells (ILCs), and NK cells [20]. Furthermore, prophylactic and immunotherapeutic treatment with HKCC demonstrated strong efficacy against respiratory pathogens (SARS-CoV-2, influenza virus, *Mycobacterium tuberculosis* and *Mycobacterium avium*) in animal models [21]. In this study, we have investigated the effects of HKCC and HKCC-treated human peripheral blood mononuclear cell (PBMC) supernatants, with and without HCV DAAs (ribavirin, telaprevir), on the inhibition of hepatitis viruses using in vitro infection models, an HCV-1a replicon system, and HBV HepG 2.2.15 cells to understand the potential of HKCC-induced human immune responses in an ex vivo system. Our results demonstrated that HKCC-stimulated various cytokines from human PBMCs, and collected supernatants significantly inhibited HCV and HBV replication, prevented HCV viral relapse, and suppressed the production of HBsAg and HBeAg without inducing any cytotoxic effects in their respective HCV-1a replicon and HepG 2.2.15 cells (Figure 1, Figure 2, Figure 3, Figure 4, Figure 5, Figure 6 and Figure 7). In conclusion, HKCC can provide an effective immunotherapy against HCV and HBV infections.

## 2. Materials and Methods

### 2.1. Heat-Killed Caulobacter crescentus (HKCC)

*Caulobacter crescentus* (*Cc*) (ATCC), was grown at room temperature (25 °C) in a PYE medium. Colony forming units (CFU) were determined by measuring optical density (O.D.) for growing *Cc* cultures, and logarithmically growing cultures were centrifuged (6000 rpm for 15 min) and stored at 4 °C, in PBS. Next, live *Cc* was heat-inactivated at 60–80 °C for 60 min and resuspended in PBS or the appropriate cell culture medium, prior to use. To ensure quality control and reproducibility, complete bacterial inactivation was confirmed by plating serially diluted HKCC suspensions onto PYE agar plates, and all batches yielded no CFU counts, confirming 100% inactivation and zero residual viability, stored at 4 °C and checked for physical stability visually, ensuring no visible auto-aggregation or precipitation, prior to use. Endotoxin levels in our working dilutions were evaluated using a standard Limulus Amebocyte Lysate (LAL) assay and were determined to be below 0.05 EU/mL ensuring that the observed antiviral effects are a specific response to the structured bacterial components rather than toxic endotoxin contamination.

### 2.2. In Vitro PBMC Stimulation

Human blood samples (*n* = 12) were obtained from normal healthy donors (50% males and 50% females, aged 20–60 years old), after informed consent. Studies with human blood samples were approved by the Health Research Ethics Board at the University of Alberta (Pro 0085358, current 27 January 2026–27 January 2027), and all research was performed in accordance with the relevant guidelines and regulations. From human blood samples, PBMCs were isolated using the Ficoll-Paque density gradient centrifugation method (Amersham Biosciences), and the interface layer, also called the buffy coat, was collected. From each donor, PBMCs (4 × 10^6^ cells/mL) were cultured in AIM V medium with various stimulants, including HKCC (5 × 10^7^ CFU/mL), PolyI:C (1 μg/mL), or PBS, in separate cultures, for 24 h at 37 °C in 5% CO_2_. Next, supernatants were collected, and frozen at 60–80 °C for long-term storage.

### 2.3. Cytokine Enzyme-Linked ImmunoSorbent Assay (ELISA)

Supernatants were tested for IFNγ, TNFα, IL-1β, IL-6, IL-10, IL-12, IL-17A, and IL-22 using commercially available human ELISA kits (Thermo Fisher Scientific; Waltham, MA, USA). Cytokine ELISAs were performed according to manufacturer’s instructions. Absorbance was measured using a DTX880 Plate Reader (Beckman Coulter; Brea, CA, USA), at 450 nm. O.D. values were interpolated with assay-specific standard curves to determine cytokine concentration. Results were reported as mean ± standard error mean (SEM) of four donor supernatants.

### 2.4. HCV-1a Replicon Assay

Anti-HCV activity was determined in HCV-1a subgenomic replicon cells (kind gift from Dr. Charles Rice, Rockfeller University, USA) as described previously [22,23,24]. Briefly, Huh7 cells (1 × 10^5^ cells/mL) containing HCV-1a replicons were plated in 24-well plates in G418-containing DMEM selection medium. The next day, cells were incubated with various direct-acting antivirals [DAAs: telaprevir (1 μg/mL), ribavirin (5 μg/mL), nucleoside analog (2′-fluoro-2′-C-methylcytidine) (0.75 μg/mL), peg-IFNα (100 IU/mL), HKCC (1 × 10^6^ CFU/mL and 1 × 10^7^ CFU/mL), or HKCC (1 × 10^7^ CFU/mL)-stimulated PBMC supernatants at various dilutions (12.5–50% of the total culture medium), at 37 °C in 5% CO_2_. These treatments were given once every 4–5 days, for 1–5 treatment cycles. For the combination treatments, cells were treated with PBMC supernatants in the presence or absence of DAAs at the stated concentrations.

For rebound experiments, after the given number of treatments, the remaining cells were split into new plates and left untreated for 1–3 rebound periods (4–5 days for each rebound period). Parallel cultures were set up to perform crystal violet staining at the end of treatment and rebound periods. In the rebound experiments (~35 days), the whole experiment was conducted in the presence of G418 antibiotic. In groups with significant inhibition of HCV RNA (marked suppression of HCV RNA), cells lacking replicons are expected to die due to the absence of resistance genes and the cytotoxic effect of G418.

### 2.5. Crystal Violet Staining Assay

Following treatments, the culture medium was removed, and the monolayers were gently washed three times with PBS to completely eliminate detached, dead cells. The remaining adherent cells were fixed and stained using 100 μL of 0.1% Crystal Violet solution for 20 min at room temperature. Excess dye was removed by washing three times with deionized water, and the plates were air-dried. The bound dye was then solubilized in 100 μL methanol, and the absorbance was measured at 570 nm using a DTX880 Plate Reader (Beckman Coulter; CA, USA).

### 2.6. Detection of HCV RNA Using Real-Time Polymerase Chain Reaction (RT-PCR)

Total cellular RNA was extracted using an RNAeasy-96 kit (Qiagen; Valencia, CA, USA), cDNA was synthesized using an iScript cDNA synthesis kit (BioRad; Hercules, CA, USA) and the copy number of HCV RNA was determined using a quantitative-PCR assay, with SYBR green dye (BioRad; CA, USA). The primers used for PCR assays were HCV UTR F: 5′-CTG TCT TCA CGC AGA AAG CG-3′, HCV UTR R: 5′-CAC TCG CAA GCA CCC TAT CA-3′, β-actin F: 5′-CGA TGC AGA AGG AGA TCA CTG-3′, β-actin R: 5′-CGA TCC ACA CGG AGT ACT TG-3′.

### 2.7. HepG 2.2.15 Cell Culture and Treatment

HepG 2.2.15 cells (5 × 10^4^ cells/mL) (obtained from Dr. DLJ Tyrrell, University of Alberta, Canada) were plated in 24 well plates in DMEM medium containing 10% FBS and 1% Pen-Strep [24]. The medium was changed every 2–3 days and replaced with freshly prepared supernatants (12.5–50% of the total culture medium) from PBS or HKCC (1 × 10^7^ CFU/mL)-stimulated PBMCs, at 37 °C in 5% CO_2_, for 7 days (total 3 treatment cycles). Adefovir (0.2 μg/mL) was used as a DAA positive control. On days 3, 5, and 7, supernatants were collected to detect the production of HBsAg and HBeAg using ELISA, and extracellular HBV DNA using PCR.

### 2.8. Detection of HBsAg and HBeAg Using ELISA

The production of HBsAg and HBeAg were measured using ELISA kits (ImmunoDiagnostics, Hong Kong, China). Briefly, 96-well plates were coated with a monoclonal antibody for either HBsAg or HBeAg. Next, day 5 and 7 supernatant (12.5–50% of the total culture medium) samples were added and incubated for 30 min. After the incubation, plates were washed five times, and an HRP-conjugated antibody specific for HBsAg or HBeAg was added to the appropriate plate and incubated for 30 min. Next, plates were washed five times, and incubated with TMB (3,3′,5,5′-tetramethylbenzidine) for color development. O.D. values were acquired using a DTX880 Plate Reader (Beckman Coulter; CA, USA), at 450 nm.

### 2.9. Detection of Extracellular HBV DNA Using RT-PCR

HBV DNA was extracted from culture supernatant using a high pure viral nucleic acid isolation kit (Roche; Basel, Switzerland) according to manufacturer’s instructions. Briefly, 200 μL of culture supernatant, 50 μL of protein kinase K, 4 μL of poly A, and 200 μL of binding buffer reagent were incubated for 10 min at 72 °C. After protein kinase K digestion and washing with buffer solution, DNA was eluted in 50 μL of elution buffer provided. Next, HBV DNA was quantified by fluorescent RT-PCR. The HBV probe and primers are forward primer HBV-F3, 5′-GGCCATCAGCGCATGC-3′, and reverse primer HBV-R3M3, 5′-C/i5Nitlnd/GCTGCGA GCAAAACA-3′, and probe HBV-P3, 5′-6-FAMCTCTGCCGATCCATACTGCGGAACTC-ZEN-3′. The PCR mix (10 μL each reaction) contained universal master mix (5 μL), primers HBV-F3 and HBV-R3M3 (each at 900 nmol/reaction), probe HBV-P3 (250 nmol/reaction), 2 μL of DNA sample and 1.5 μL of RNase/DNase-free water. RT-PCR cycle: after 2 min at 50 °C and 10 min at 95 °C, there were 45 cycles (95 °C for 15 s and 60 °C for 1 min) of PCR amplification for HBV detection. RT-PCR was performed using an ABI Prism 7900HT SDS (Applied Biosystem; Waltham, MA, USA).

### 2.10. Standard Curve for HBV DNA Using a pKSV-HBV1 Plasmid

The pKSV-HBV1 plasmid was used to prepare the standard curve for the RT-PCR assay for detecting extracellular HBV DNA. This plasmid contains a tandemly arranged HBV 991 DNA inserted in a pKSV vector, which was propagated in *Escherichia coli* (*E. coli*), and purified using the QIAGEN Plasmid Midi Kit (QIAGEN; NY, USA) [24]. The purified plasmid DNA was digested to generate linear full-length HBV strands using endonuclease XbaI (Invitrogen; Carlsbad, MA, USA) and the insert size was confirmed by 0.8% agarose gel electrophoresis (1 Kb size quick load DNA ladder). The pKSV HBV DNA was quantified by A_260_ measurement using a NanoDrop Spectrophotometer (Thermo Fisher Scientific; MA, USA), and the copy number was calculated based on the HBV 991 DNA molecular weight (3221 bp). For each experiment, a standard curve ranging from 10^1^ to 10^8^ copies/mL of linearized pKSV-HBV1 DNA was prepared.

### 2.11. HepG 2.2.15 Cytotoxicity Assay Using XTT Reagent

In a 96-well plate, HepG 2.2.15 cells (2 × 10^4^ cells/well) were seeded and left overnight, at 37 °C in 5% CO_2_, to allow cells to attach to the bottom of the well. Next day, cells were treated with Adefovir (0.2–5 μg/mL), and PBS- and HKCC (1 × 10^7^ CFU/mL)-stimulated PBMC supernatants (diluted at 5–50% of the total medium), and incubated for 72 h, at 37 °C in 5% CO_2_. After the incubation, plates were washed with pre-warmed saline and supplemented with fresh DMEM medium. Next, 50 μL of tetrazolium salt 2,3-bis-(2-methoxy-4-nitro-5-sulfophenyl)-2H-tetrazolium-5-carboxanilide (XTT) was added to each well, and incubated for 2 h, at 37 °C in 5% CO_2_. Plates were read using a DTX880 Plate Reader (Beckman Coulter; CA, USA), at 450 nm.

### 2.12. Graph and Statistical Analysis

Graphical and statistical tests were generated and performed using GraphPad Prism Software 10.2.0 (GraphPad; Boston, MA, USA). No data points were excluded from the analysis. Statistical differences were determined using one-way analysis of variance (ANOVA), followed by a Šídák post hoc test for multiple comparisons. Prior to ANOVA testing, assumptions of normality and equal variances were verified. *p* ≤ 0.05 was used to determine significance.

## 3. Results

**Heat-killed *Caulobacter crescentus* (HKCC)-induced multi-functional cytokines in human PBMCs, and its supernatant inhibited HCV RNA replication in HCV-1a replicon cells.**

Human PBMCs (4 × 10^6^ cells/mL) were isolated from healthy donors and stimulated with PBS, HKCC at 1 × 10^7^ CFU/mL, or HKCC at 5 × 10^7^ CFU/mL for 24 h. Supernatants were collected and analyzed for production of various cytokines. HCV-1a replicon cells were treated with HKCC directly or with supernatants from PBMCs to determine if HCV RNA replication could be inhibited. Human PBMCs stimulated with HKCC at 5 × 10^7^ CFU/mL showed a significant increase in IFNγ, TNFα, IL-22, IL-17, IL-12, IL-10, IL-6, and IL-1β production, compared to PBS (Figure 1A). Human PBMCs treated with PBS did not induce any cytokines (Figure 1A).

Direct treatment of HKCC at 1 × 10^6^ CFU/mL and 1 × 10^7^ CFU/mL showed no inhibitory effects on HCV RNA replication in HCV-1a replicon cells (Figure 1B). Interestingly, supernatants from human PBMCs stimulated with HKCC demonstrated a significant reduction in HCV RNA replication compared to the medium control (Figure 1C). Notably, human PBMC supernatants stimulated with HKCC at 1 × 10^7^ CFU/mL, were the most effective at reducing HCV RNA replication in comparison to all other groups tested (Figure 1C). However, the degree of the viral suppression was correlated positively with the doses (1 × 10^6^ vs. 1 × 10^7^ CFU/mL) of HKCC. HCV has been shown to impair TLR-3 signaling, which accounts for HCV persistence in the liver. Interestingly, supernatants from Human PBMCs stimulated with HKCC had remarkably higher reduction in HCV RNA as compared to polyI:C, a surrogate for double-stranded RNA and a TLR-3 agonist (Figure 1C).

Altogether, these results highlighted HKCC’s potential as a host-direct therapeutic (HDT) against HCV.

**Combination treatment with HKCC-stimulated PBMC supernatants enhanced the inhibitory effects of DAAs on HCV RNA replication and viral rebound in HCV-1a replicon cells without inducing cytotoxic effect on host cells.**

In combination studies, we assessed the inhibitory effects of HKCC-stimulated human PBMC supernatants in conjunction with DAAs, ribavirin (an HCV polymerase inhibitor) and telaprevir (an HCV protease inhibitor) on HCV RNA replication in 2 and 3-drug combinations.

First, we assessed the effects of a single treatment on HCV viral RNA replication. In the two-drug combination studies, one treatment cycle of telaprevir alone, telaprevir + ribavirin, HKCC-stimulated human PBMC supernatants with ribavirin as well as telaprevir led to significant reduction in HCV RNA replication compared to the ribavirin and telaprevir groups (Figure 2). Notably, in three-drug combination studies, HKCC supernatants provided much higher inhibition of HCV RNA replication to below detection levels when combined with both ribavirin + telaprevir compared to both DAA combination groups (Figure 2). After two treatments, supernatants collected from HKCC-stimulated PBMCs further enhanced antiviral effects in combination with ribavirin and telaprevir in both 2- and 3-drug combinations (Figure 3A), as compared to the single treatment (Figure 2). Next, we investigated the combination effect of HKCC-stimulated PBMC supernatants with both HCV polymerase and protease inhibitors (DAAs) upon multiple treatments. In these experiments, in addition to ribavirin and telaprevir we also included pegylated interferon alpha (peg-IFNα), which is used to treat chronic HCV infection as a host-directed therapy. Comparing HKCC-stimulated PBMC supernatants verses peg-IFNα alone, and/or in combination with the DAA treatments, HKCC-stimulated supernatants exhibited much superior inhibition of HCV RNA replication in all combinations (Figure 3A–C). Likewise, similar trends were observed in the combination treatments with DAAs (ribavirin, telaprevir, ribavirin + telaprevir) or peg-IFNα and HKCC-stimulated PBMC supernatants after 4 and 5 treatment cycles (Figure 3B,C). Since immunity is weaker during antiviral treatments than without the treatment, drug discontinuation may lead to an increase in viral load. HCV has high genetic variability and resistance-associated amino acid changes may lead to future failure of currently available DAA treatments. Therefore, next, we investigated the efficacy of HKCC-stimulated PBMC supernatants in controlling HCV viral resurgence. After five treatment cycles, subsequent HCV-1a replicon cell cultures were left untreated for one or three rebound periods, with each rebound period consisting of 4–5 days, after which HCV RNA levels were quantified. In the third rebound experiments, telaprevir alone, telaprevir + ribavirin, and HKCC-stimulated human PBMC supernatants alone significantly blunted viral rebound compared to the one rebound period (Figure 4). However, combination treatment of HKCC-stimulated human PBMCs supernatants with DAAs demonstrated significant attenuation of viral resurgence (Figure 4). Notably, treatment with HKCC-stimulated human PBMC supernatants in conjugation with telaprevir, and telaprevir + ribavirin retained marked attenuation of HCV RNA replication after the 3rd rebound period, whereas with peg-IFNα treatment in conjunction with ribavirin or ribavirin + telaprevir virus flared up. These results are indicative of marked suppression of HCV infections with a novel immunotherapeutic HKCC in combination with HCV polymerase and protease inhibitors (Figure 4).

Antiviral potential of the HKCC-stimulated supernatants was also investigated with a new generation of HCV-specific viral RdRp nucleoside inhibitor (2′-fluoro-2′-C-methylcytidine) for HCV RNA inhibition and viral rebound (Figure 5). Two treatment cycles of the nucleoside analog alone showed no significant reduction in HCV RNA replication or attenuation of viral rebound (Figure 5). In contrast, the addition of supernatants from human PBMCs stimulated with HKCC resulted in a significant reduction in HCV RNA replication, and attenuation of viral resurgence compared to the nucleoside group (Figure 5).

We also validated that the inhibitory effects of HKCC-stimulated human PBMC supernatants reflected a reduction in HCV-1a replicon activity, rather than cell death caused by the treatment groups. HCV-1a replicon cells and its parent cell line that lacks the HCV-1a replicon, Huh7 cells were treated with medium, various DAAs alone, and/or in combination of HKCC-stimulated PBMC supernatants in parallel cultures. After four treatment cycles and one rebound period, cells were stained with crystal violet to assess HCV-1a replicon activity in HCV-1a replicon cells and the cytotoxic effect on Huh7 cells. In the HCV-1a replicon activity assay, medium and DAAs (telaprevir, ribavirin, and nucleoside analog) alone were stained strongly by crystal violet (Figure 6A). Whereas treatment with HKCC-stimulated PBMC supernatants alone and in combination with DAAs (HKCC + telaprevir, HKCC + ribavirin and HKCC + nucleoside analog) showed significantly fewer cells stained with crystal violet—suggesting strong reduction in HCV-1a replicon activity (Figure 6A). However, in the Huh7 cell cytotoxicity assay, all treatment groups showed strong staining for crystal violet, suggesting there was little-to-no cytotoxic effect of any treatment on the Huh7 cells (Figure 6B). Altogether, these results showed that treatment with HKCC-stimulated human PBMC supernatants alone and in combination with both HCV polymerase and protease inhibitors (DAAs) reduced HCV1a replicon activity without inducing any cytotoxic effects, and further affirmed the HCV RNA results from previous experiments (Figure 2, Figure 3, Figure 4 and Figure 5). Interestingly, visually we noted that there was a further reduction in HCV1a replicon activity after a rebound period of 4–5 days which could be attributed to the activation of host antiviral activity and/or restoration of the immune dysfunction by HKCC (Figure 6A).

Overall, the effectiveness of DAA treatments against HCV infections, drug resistance and averting re-infections, can be improved with host-direct immunotherapeutic HKCC.

**Supernatants from HKCC-stimulated human PBMCs significantly inhibited the production of extracellular HBV DNA, HBsAg, and HBeAg from HepG 2.2.15 cells, without any cytotoxicity.**

HepG 2.2.15 cells (5 × 10^4^ cells/mL) were treated with human PBMC supernatants stimulated with medium, PBS, or HKCC (1 × 10^7^ cfu/mL), every 2–3 days, for 7 days (totaling three treatment cycles). Adefovir was included as a DAA (against HBV) positive control. It is a nucleotide analog and used to treat chronic (long-term) HBV infection as a chemotherapeutic drug. It is effective against both wild-type as well as lamivudine-resistant HBV. After each treatment cycle culture supernatants were collected to determine the production of extracellular HBV DNA, HBsAg, and HBeAg (Figure 7A–C). HBV DNA, HBsAg and HBeAg are critical markers to monitor infection status and have been used to determine the efficacy of various agents for the treatment of chronic HBV. High levels of HBsAg indicates the presence of HBV. The presence of HBeAg (extracellular form of core antigen) indicates high and active viral replication in hepatocytes. It has been claimed that HBeAg suppresses host immune response, thereby contributing to persistent chronic infection. Positivity of the HBeAg has been shown to be associated with an increased risk of hepatocellular carcinoma (HCC). We also assessed the cytotoxic effects of each treatment on HepG2 2.2.15 cells, using an XTT assay (Figure 7D). HKCC-stimulated human PBMC supernatants, tested at 50% and 25% of total culture medium, demonstrated a significant reduction in extracellular HBV DNA production, HBsAg secretion, and HBeAg release compared to the medium and PBS treatments (Figure 7A–C). Although, after three treatments compared to HKCC supernatant, adefovir, an HBV DNA polymerase inhibitor, showed higher inhibition of extracellular HBV DNA, HKCC supernatants exhibited similar HBsAg inhibition and much superior inhibition of HBeAg than adefovir. However, some of the effects of adefovir on HBV DNA could not be ruled out due to its cytotoxicity. These results support the immunotherapeutic potential of HKCC in activating host antiviral immune responses. HKCC-stimulated human PBMC supernatants had no cytotoxic effects on HepG 2.2.15 cells, which were similar to the medium and PBS treatments (Figure 7D). In contrast, Adefovir demonstrated greater cytotoxicity under the experimental conditions tested. All in all, these results showed the potential application of HKCC in HBV infections and appeared well tolerated in the in vitro system.

## 4. Discussion

In this study, we demonstrated the ability of HKCC to act as an innate immune modulator that can serve as an effective host-based immunotherapeutic against HCV and HBV infections. Stimulation of human PBMCs with HKCC induced the production of various cytokines, including IFNγ, TNFα, IL-22, IL-17, IL-12, IL-10, IL-6, and IL-1β (Figure 1A). Furthermore, HKCC-stimulated human PBMC supernatants significantly inhibited HCV RNA replication and attenuated HCV viral relapse in HCV-1a replicon cells with strong synergistic effects in combination with HCV polymerase and protease inhibitors (Figure 2, Figure 3, Figure 4, Figure 5 and Figure 6). In addition, these supernatants inhibited HBV DNA production, secretion of HBsAg, and release of HBeAg in HepG 2.2.15 cells (Figure 7). Therefore, HKCC induces antiviral immune responses effective against both RNA and DNA viruses. In HCV/HBV co-infections, treatment with current DAAs for HCV have shown to reactivate HBV, leading to serious liver problems including HCC and cirrhosis [12]. Considering the potent antiviral effects of HKCC against both viruses, it can be implemented as a possible host-based immune-therapeutic that will complement current DAA treatments against HCV infections and simultaneously suppress HBV reactivation.

Our earlier studies with HKCC demonstrated its ability to effectively stimulate the innate immune system through the upregulation of antigen-presenting functions, the expression of co-stimulatory molecules, and the production of effector cytokines in macrophages, dendritic cells (DCs), granulocytes, and ILCs with excellent preventive and therapeutic effects against SARS-CoV-2, influenza virus and *Mycobacterium tuberculosis* in animal models [20,21]. In the current study, we show the potential of HKCC to induce human immune responses in an ex vivo system using HKCC-treated human PBMCs from healthy donors. HKCC-stimulated secretion of various cytokines including IFNγ, TNFα, IL-22, IL-12, IL-6, and IL-1β from human PBMCs are relevant in controlling HCV and HBV infections (Figure 1A). The antiviral effects of HKCC are driven by a collective network of cytokines working in parallel, with each contributing to a unique mechanism of viral inhibition, hepatic regeneration and repair. Mechanistically, multiple HKCC-induced cytokines are associated with suppression of HBV and HCV viral replication, engaging with multiple parallel signaling pathways within the hepatocytes. The upregulation of IFNγ and IL-6 drives the classic JAK-STAT pathway, activating STAT1 and STAT3 homodimers to induce potent antiviral interferon-stimulated genes (ISGs) that actively degrade HCV-1a replicon RNA, block protein translation, and inhibit HBV pre-genomic RNA encapsulation [13,22]. Concurrently, TNFα and IL-1β activate the NF-kB and MAPK cascades, which restrict HBV by epigenetically silencing the cccDNA transcription template and destabilizing viral nucleocapsids [13,25]. The secretion of IL-12 acts as a critical upstream bridge capable of amplifying these pathways, facilitating the reversal of immune tolerance, reactivating exhausted immune responses, and reinforcing a localized Th1-polarized immune response [26]. Moreover, IL-22 selectively binds hepatocyte IL-22R1 to activate anti-apoptotic pathways (such as Bcl-2), preserving cell viability and counteracting any localized inflammatory cytotoxicity, induced by high levels of TNFα and IL-1β [27]. This diverse cytokine network acts cooperatively, targeting distinct stages of the HBV and HCV life cycles while maintaining hepatocyte homeostatic survival. Regarding host-based approaches, TLR-7, TLR-8, and RIG-I agonists have been tried but were largely unsuccessful due to severe inflammation and exacerbation of liver disease [28,29,30,31,32]. Comparatively, in vivo studies of HKCC have consistently shown induction of a balanced immune response that effectively protects against foreign invaders while minimizing tissue damage [20,21]. Altogether the cytokine profile induced by HKCC from human PBMC donors demonstrates an immunomodulatory effect that can be beneficial in controlling HCV/HBV infections and protecting hepatocytes and liver from hepatitis virus-induced damage.

Although 12–24 weeks of treatment with direct-acting antivirals can cure >95% of a person’s HCV infection, access to such diagnosis and treatment is low. Furthermore, high costs, adherence to a regime of treatment, re-infection risks and limited ability to reverse existing liver damage remain significant challenges. With all the HCV DNA polymerase and protease inhibitors used, the addition of HKCC-stimulated human PBMC supernatants significantly enhanced their anti-HCV effects with low or no viral rebound after five (4–5 days interval) treatments (Figure 2, Figure 3, Figure 4, Figure 5 and Figure 6). This suggests that a sustainable virological response was induced that was measurable even after cessation of treatment (Figure 6). Notably, the combination treatment of HKCC-stimulated human PBMCs supernatants with telaprevir (HCV protease inhibitor) and telaprevir + ribavirin (targeting both protease and polymerase) markedly inhibited HCV replication and showed no signs of viral relapse, indicating significant attenuation of viral rebound of HCV (Figure 4 and Figure 6). Furthermore, in comparison with peg-IFNα combinations (IFNα + ribavirin, IFNα + ribavirin + telaprevir), the addition of HKCC-stimulated human PBMC supernatants to both DAAs exhibited much higher anti-HCV effects without viral flare up, indicating HKCC can complement the DAAs in suppressing HCV replication and blunting viral relapse (Figure 2, Figure 3 and Figure 4). Our data suggested that HKCC, as a host-based immunotherapeutic, can be a promising treatment against HCV to reduce costs, the duration of treatments, and can prevent reinfection and liver damage.

Although approved antiviral nucleos(t)ide analogs can effectively suppress HBV replication, they do not fully clear the virus from infected cells. As a result, resistant mutants develop against them upon long-term use. Also, patients with chronic HBV are administered lifelong nucleos(t)ide treatments to prevent viral flare-up and mitigate the risk of severe liver diseases [33,34]. The clinical application of the immunomodulator IFNα is restricted by concerns regarding its safety and the severity of associated adverse reactions, rendering long-term administration challenging. Other cytokines such as TNFα, IL-6, IL-1β have also been shown to control HBV replication and contribute to HBV cure in different models [25]. In a recent study, HBV entry was inhibited up to 90% in IL-6-pre-treated cells resulting in a strong reduction in cccDNA and HBsAg secretion. Both IFNγ and TNFα interfered with cccDNA integrity and stability in HBV-infected cells [25]. Blockage of IFNγ and TNFα abrogated antiviral effects of T cells [15,25]. IL-1β was found to have a very potent antiviral effect against HBV in vitro [25]. IL-12 promoted cellular immunity and modulates the cytotoxic activity of CTLs and NK cells and enhances antiviral properties. Elevated IL-12 rescues the antiviral function of exhausted HBV-specific CD8^+^ T cells [25]. As described in Figure 1A, HKCC induced secretion of multi-functional cytokines from human PBMCs and their supernatants remarkably inhibited HBV DNA, HBsAg and HBeAg when added to the HBV infected cells (Figure 7). No cytotoxicity was observed in uninfected hepatocytes in contrast to the nucleotide analog adefovir (Figure 7D). Therefore, concurrent or sequential treatment with a novel immunomodulator like HKCC, in conjunction with HBV DAAs may enhance the suppression of HBV viral replication and lower HBV reactivation rates.

In HCV/HBV co-infected patients, HCV replicates and activates IFN genes to produce IFNs that impose an anti-viral effect on HBV [11]. With effective DAA treatments against HCV, the HCV infection is cleared, and the anti-viral effects on HBV are lifted, reactivating HBV [11]. Clinical reports indicated that the HBV reactivation rate was 8% with the current HCV DAA treatments, and 49% with the peg-IFNα + ribavirin treatment [11,12,22,23,35]. To mitigate the risk of HBV reactivation, DAA treatments against HCV need to be supplemented with therapeutics that suppress HBV activity. Moreover, considering the adverse effects of a six-month long peg-IFNα regimen, including bone marrow depression, flu-like symptoms, psychiatric disorders, and autoimmune diseases—a better-tolerated therapeutic is needed [36]. In a head-to-head comparison between host-based therapeutic peg-IFNα (that has been in clinical use for a long time), HKCC-stimulated human PBMC supernatants alone and in combination with DAAs (ribavirin, telaprevir, and telaprevir + ribavirin), demonstrated significantly better inhibition of HCV RNA replication and suppression of viral relapse, compared to the peg-IFNα alone and its combinations with respective DAAs (Figure 3 and Figure 4). Intriguingly, HKCC-stimulated human PBMC supernatants also inhibited HBV DNA production, HBsAg secretion, and HBeAg release, without any cytotoxic effects on HepG 2.2.15 cells (Figure 7A–D). Altogether, HKCC can be implemented with HCV and HBV DAAs as a safer, more effective alternative to peg-IFNα.

Based on our current ex vivo study, HKCC has the potential to complement DAAs to treat chronic HCV and HBV infections. Looking forward to translational applications, HKCC could be developed as a promising host-based therapeutic to reduce dosage, duration of treatment, and enhance the therapeutic efficacy of current DAAs. Moreover, with chronic hepatitis infections, managing the delicate immunological balance between systemic immune activation and inflammation will necessitate precise dosing regimens of HKCC and potentially complementing DAAs to minimize adverse side effects. Notably, HKCC induces a potent innate immune response, with no inherent risk of systemic inflammation. Preclinical studies of other viral and bacterial infection models have established that oral or intranasal administration of HKCC effectively reduces viral and bacterial loads while demonstrating an excellent safety profile with no adverse immunopathologies [36]. Specifically, HKCC has been tested as an immunotherapy against SARS-CoV-2, influenza virus, and *Mtb* and *Mav*, highlighting its ability to control and resolve active infections [21]. Oral and intranasal administration of HKCC, prophylactically and therapeutically, led to a significant reduction in viral and bacterial loads in lungs, as well as bacterial loads in liver and spleen suggesting that HKCC can induce protective immune responses in the liver and be beneficial against HCV and HBV infections [21]. Moreover, we utilized the HCV-1a replicon system and HBV HepG2.2.15 cells. These models are robust, well-established standards for measuring direct, intracellular viral replication kinetics and host interferon-stimulated gene (ISG) responses within hepatocytes. However, they operate in the absence of critical liver-resident immune populations—such as Kupffer cells, hepatic stellate cells, liver-sinusoidal endothelial cells (LSECs), and infiltrating NK/T cells [37]. Considering HKCC’s ability to influence innate immune cells, in an in vivo setting HKCC would positively impact the complex hepatic microenvironment by activating antiviral immune responses, promoting tissue repair and regenerative processes, and enhancing therapeutic efficacy and safety [20]. For future studies, the specific safety profile and optimal dosing of HKCC in animal models of hepatitis (such as HBV transgenic mice or humanized liver models) are the subject of our ongoing and future investigations, recent preclinical literature has strongly validated the baseline safety of HKCC. Furthermore, the cytokines induced by HKCC and their direct effect on the clearance of the HCV-1a replicon and HBV in HepG2.2.15 cells remain to be fully mapped. Therefore, for future studies, we are looking to perform cytokine neutralization assays, receptor blockade studies, and pathway inhibition approaches. Beyond the HKCC-stimulated PBMC cytokine response, it is important to understand activated PBMCs may secrete a variety of other bioactive molecules, including microRNAs (miRNAs) that modulate antiviral immunity. For example, upregulation of miR-146a/miR-155 has been associated with promoting HCV infection and dysregulation of liver metabolism leading to worsening disease outcomes by interfering with TLR7 signaling [38]. Therefore, future studies investigating the role of HKCC on miRNA will provide further insight into its antiviral effects on HBV and HCV infections.

## 5. Conclusions

In conclusion, our findings demonstrate that HKCC functions as a potent host-directed immunomodulator, coordinating a multi-functional cytokine response that is associated with effectively suppressing intracellular HBV and HCV-1a replication, restricting viral shedding, and blocking post-treatment viral relapse. From a translational perspective, these data suggest that HKCC holds significant potential as an adjunct therapy, which could be combined with standard direct-acting antivirals or nucleos(t)ide analogues to reduce viral antigen burden, and mitigate the risk of rebound. However, a major limitation of this study is its strictly in vitro and ex vivo framework, meaning these baseline hepatocyte observations operate in the absence of a multicellular hepatic immune microenvironment or living circulatory system. Consequently, while these preliminary efficacy data are highly promising, rigorous downstream in vivo preclinical evaluation in specialized animal models remains a mandatory next step to characterize systemic safety profiles and optimize therapeutic dosing ranges before advancing toward human clinical application.

## Data Availability

The original contributions presented in this study are included in the article. Further inquiries can be directed to the corresponding author.
